# Accuracy gains from conservative forecasting: Tests using variations of 19 econometric models to predict 154 elections in 10 countries

**DOI:** 10.1371/journal.pone.0209850

**Published:** 2019-01-10

**Authors:** Andreas Graefe, Kesten C. Green, J. Scott Armstrong

**Affiliations:** 1 Macromedia University, Munich, Germany; 2 University of South Australia Business School, Adelaide, Australia; 3 The Ehrenberg-Bass Institute, University of South Australia, Adelaide, Australia; 4 Wharton School, University of Pennsylvania, Philadelphia, PA, United States of America; University of Toronto, Rotman School, CANADA

## Abstract

**Problem:**

Do conservative econometric models that comply with the Golden Rule of Forecasting provide more accurate forecasts?

**Methods:**

To test the effects of forecast accuracy, we applied three evidence-based guidelines to 19 published regression models used for forecasting 154 elections in Australia, Canada, Italy, Japan, Netherlands, Portugal, Spain, Turkey, U.K., and the U.S. The guidelines direct forecasters using causal models to be conservative to account for uncertainty by (I) modifying effect estimates to reflect uncertainty either by damping coefficients towards no effect or equalizing coefficients, (II) combining forecasts from diverse models, and (III) incorporating more knowledge by including more variables with known important effects.

**Findings:**

Modifying the econometric models to make them more conservative reduced forecast errors compared to forecasts from the original models: (I) Damping coefficients by 10% reduced error by 2% on average, although further damping generally harmed accuracy; modifying coefficients by equalizing coefficients consistently reduced errors with average error reductions between 2% and 8% depending on the level of equalizing. Averaging the original regression model forecast with an equal-weights model forecast reduced error by 7%. (II) Combining forecasts from two Australian models and from eight U.S. models reduced error by 14% and 36%, respectively. (III) Using more knowledge by including all six unique variables from the Australian models and all 24 unique variables from the U.S. models in equal-weight “knowledge models” reduced error by 10% and 43%, respectively.

**Originality:**

This paper provides the first test of applying guidelines for conservative forecasting to established election forecasting models.

**Usefulness:**

Election forecasters can substantially improve the accuracy of forecasts from econometric models by following simple guidelines for conservative forecasting. Decision-makers can make better decisions when they are provided with models that are more realistic and forecasts that are more accurate.

## Introduction

The evidence-based forecasting principle known as the Golden Rule of Forecasting advises forecasters to adhere closely to cumulative prior knowledge about the situation. We test whether following this principle of conservatism can help to *improve the accuracy* of econometric models’ *ex ante forecasts*. To help forecasters to apply the Golden Rule, Armstrong, Green and Graefe provided 28 guidelines for conservative forecasting, such as how to formulate a forecasting problem; how to forecast with judgmental, extrapolative, and causal methods; how to combine forecasts from different methods; and how to adjust forecasts. They then assessed the effects of each guideline on out-of-sample forecast accuracy by reviewing published studies that compared the accuracy of forecasts from conservative and non-conservative forecasting methods. Of the 105 studies they identified, 102 supported the guidelines. On average, *ignoring* a guideline increased forecast error by more than 40% [1]. Further research on the Golden Rule produced additional evidence and a revision of the guidelines [2]. Among the changes was a suggestion to use knowledge models as an alternative to regression analysis. The aim of knowledge models is to include all variables that are known to have important causal relationships with the subject of the forecast, based on the domain knowledge of experts and evidence from experimental studies. The latest version of the Golden Rule is available at ForPrin.com.

This paper tests the effect of following conservative guidelines on the accuracy of forecasts from published models originally estimated using multiple regression analysis. In particular, we tested three of the guidelines on 19 regression models used to forecast vote shares in 154 elections in ten countries.

## Econometric models for forecasting elections

The development of causal models for forecasting voting in elections has become an important sub-discipline of political science. As of September 2018, about 2,000 results were identified by a Google Scholar search for the two terms “election forecasting” and “model.” Evidence on the models’ predictive validity should be of interest to researchers whose theories of voting behavior are represented by the models, and to decision-makers whose plans vary depending on their expectations of who will win an election.

Causal theories to which the modelers ascribe identify influences on voting behavior; election forecasting models include variables that represent these influences. Most election forecasting models represent the theory of retrospective voting, which views an election as a referendum on the incumbent government’s performance, often based on the country’s economic performance. Thus, retrospective voting theory assumes that voters reward the incumbent party for good performance and punish it otherwise. Causal models typically represent this theory by using changes in one or more macroeconomic variables—such as GDP, unemployment, or prices—to measure performance. The models often include popularity poll-based variables as proxies for voters’ satisfaction with the government’s handling of both economic and non-economic issues.

Many of the models include variables that represent aspects of the country’s electoral system affecting voting behavior or historical patterns of voting behavior. For example, the time the incumbent party has held power can be used to allow for the observation that, historically, leaders have often enjoyed a “honeymoon” period of popularity following their first election, with the effect fading through a leader’s tenure as the electorate’s desire for change increases.

In the U.S., political economy models have been established in presidential election seasons since the late 1970s [3]. For the seven elections from 1992, political scientists and economists have published their models and forecasts prior to the election in special sections of scientific journals including *Political Methodologist* 5(2), *American Politics Research* 24(4) and *PS*: *Political Science and Politics* 34(1), 37(4), 41(4), 45(4), and 49(4). That work also spearheaded the development of election forecasting models in other countries, many of which featured in two special issues of the *International Journal of Forecasting* 26(1) and 28(4). In particular, researchers have developed models for France, Germany, the U.K., Portugal, Spain, Turkey, Australia, and Japan. The models have been used to test theories of voting and to estimate the relative effects of individual variables on the aggregate popular vote. Most importantly for this paper, they have been used to provide *ex ante* forecasts of election outcomes, typically many months before the election is held.

The dominant method for estimating political economy models is multiple regression analysis. Multiple regression analysis estimates variable weights that provide the least-squared-error fit to a given sample of data. The resulting variable weights are then applied to new values of the causal variables to make forecasts.

We used three criteria for including a model in our analysis. The model (1) was estimated with multiple linear ordinary least squares (OLS) regression analysis, (2) predicted national election results, and (3) was published in an academic journal. However, the forecasters of some models did not publish their data and did not respond to, or declined, our request for their data; these models were excluded from analysis.

Nineteen models from ten countries met our criteria. While those models are not exhaustive of the election forecasting literature, we believe that they do provide a representative sample of the models that have been developed for different countries. Table 1 provides an overview of the 19 models’ key features: the dependent variable, the number of elections (observations) in the estimation sample, and the number of economic and political variables in the model. The median ratio of observations to variables was five.

**Table 1:** Key features of the 19 models analyzed in this study.

		Number of	
Country / Election / Model	Dependent variable	Elections	Variables
**Australia (general)**			
Cameron & Crosby [5]	Incumbent vote	40	5
Jackman [6]	Incumbent vote	22	3
**Canada (general)**			
Bélanger & Godbout [7]	Incumbent vote	19	4
Nadeau & Blais [8]	Liberal vote	13	4
**Italy (national, European, and local)**			
Bellucci [9]	Incumbent vote	9	3
**Japan (general)**			
Lewis-Beck & Tien [10]	LDP (percent seats)	17	3
**Netherlands (legislative)**			
Dassonneville, Lewis-Beck & Mongrain [11]	Incumbent vote	20	3
**Portugal (general)**			
Magalhães & Aguiar-Conraria [12]	Incumbent vote	11	3
**Spain (general)**			
Magalhães, Aguiar-Conraria & Lewis-Beck [13]	Liberal vote	14	4
**Turkey (general)**			
Toros [14]	Incumbent vote change	11	3
**U.K. (general)**			
Lewis-Beck, Nadeau & Bélanger [15]	Incumbent vote	12	3
**U.S. (presidential)**			
Fair [3]	Incumbent vote	25	7
Cuzán [16]	Incumbent vote	25	5
Abramowitz [17]	Incumbent vote	17	3
Campbell [18]	Incumbent vote	17	2
Lewis-Beck & Tien [19]	Incumbent vote	16	4
Holbrook [20]	Incumbent vote	16	3
Erikson & Wlezien [21]	Incumbent vote	16	2
Lockerbie [22]	Incumbent vote	15	2
**Median across all 19 models**		**16**	**3**

Given the attention that election forecasting attracts in the U.S., models for forecasting U.S. presidential elections form the largest group; a total of eight models. Australian and Canadian general elections have two models each, while there is only one model each for Italy, Japan, the Netherlands, Portugal, Spain, Turkey and the U.K.

In general, the models can be written as:

$$V=a+\sum_{i=1}^{k}b_{i}x_{i}$$

where *V* is the party’s expected share of the national two-party popular vote, *a* is the vote that the party would get if all the causal variables were zero (the intercept), and the *b*_*i*_ ‘s are the coefficients—all estimated from historical data—of the *k* causal variables, *x*_*i*_ to *x*_*k*_.

## Conservative guidelines for causal models

When estimating variable weights, multiple regression analysis cannot account for uncertainty arising from sources including biases in the data, use of proxy variables, omission of important variables, inclusion of irrelevant variables, lack of variation in variable values in the estimation sample, and error in predicting or controlling causal variables in the future. As a result, multiple regression models are insufficiently conservative for forecasting as they tend to *over*fit an incomplete model specification to inadequate estimation data [4].

The Golden Rule of Forecasting provides four conservative guidelines for causal models [1]. We test three: (I) modify effect estimates to reflect uncertainty through either damping or equalizing, (II) combine forecasts from dissimilar models, and (III) include in one single model all of the causal variables used in the various available models. We hypothesized that following these guidelines would result in forecasts that were more accurate than those from models estimated using multiple regression analysis.

### Modify effect estimates to reflect uncertainty

Regression reduces the estimated effect of a variable in response to unexplained variation in the estimation data. It does not, however, compensate for all sources of uncertainty. Damping and equalizing causal variable coefficient estimates are conservative strategies that can be used to compensate for some of the residual uncertainty.

#### Damp coefficients

Damping refers to the general idea of reducing the size of an estimated effect toward having no effect. Damping has been used with extrapolation models by reducing the magnitude of an estimated trend resulting in reductions in forecast errors of about 12% [1]. The authors of that paper suggested that damping might also be useful for causal models. Following the same rationale as for extrapolation models, they concluded that the actual causal effects are weaker than those estimated from the data by regression analysis. Hence, forecasts should stay closer to the regression model’s constant. Unlike extrapolation, however, regression analysis already adjusts for uncertainty. As a result, damping is likely to be less useful when applied to regression coefficients.

Moreover, damping is a conditional guideline. It is not expected to work if the estimated coefficient is lower than what one would expect based on prior knowledge. If, on the other hand, the forecaster is uncertain over whether future causal variables values will be more extreme than those in the estimation data, the case for damping would seem stronger.

Unlike extrapolation, Armstrong, Green and Graefe were unable to find evidence on whether damping regression coefficients towards no effect improves the accuracy of *ex ante* forecasts [1]. This paper addresses the question of whether and when damping can be productively applied to multiple regression model coefficients.

Damping coefficients is not a new idea. For example, an early study tested “ridge regression”—a sophisticated approach to damping—using simulated data. Ridge regression model forecasts were more accurate than OLS model forecasts, which in turn were more accurate than equal-weights model forecasts [23]. We are not aware of any tests of the accuracy of *ex ante* ridge regression model forecasts using real data.

A simple strategy for damping is to multiply the estimated weights with a factor *d*. The “damped” version of the original regression model can be written as:

$$V=a+(1-d)\sum_{i=1}^{k}b_{i}x_{i}$$

The factor *d* can range from 0 to 1. For *d* = 0, the original regression model would remain unchanged, which means no damping. For *d* = 1, the model coefficients are in effect zero and the model forecast is simply the value of the intercept *a*—the incumbent’s vote share that would be obtained if the predictor variables were equal to their historical mean. The bigger the factor *d*, the greater is the shrinking toward the historical average incumbent vote share.

#### Equalize coefficients

Equalizing is useful if there is uncertainty about the relative importance of the causal variables; the greater the uncertainty, the more one should adjust the coefficients towards equality. When relative effect sizes are highly uncertain, one should consider the most extreme case of equalizing and assign equal-weights to all variables expressed as differences from their mean divided by their standard deviation (i.e., standardized).

To equalize, standardize the variables, estimate the model using multiple regression analysis, and adjust the estimated coefficients toward equality. The adjusted vote equation can be written as:

$$V=a+(1-e)\sum_{i=1}^{k}b_{i}x_{i}+\frac{e}{k}\sum_{i=1}^{k}b_{i}\sum_{i=1}^{k}x_{i}$$

where *e* is the equalizing factor, which can range from 0 to 1. The greater the equalizing factor *e*, the greater the amount of equalizing. An equalizing factor *e* = 0 yields the equivalent of the original multiple regression model in standardized variables. On the other extreme, when *e* = 1, all model coefficients are assigned equal-weights.

One review looked at comparative studies on equal-weights published since the 1970s in a variety of areas, and concluded that equal-weights models often provide *ex ante* forecasts that are more accurate than those from regression models [24]. For example, one of those studies analyzed the relative predictive accuracy of forecasts from regression and equal-weights models by making out-of-sample forecasts using five real non-experimental social science datasets and a large number of synthetic datasets. Regression weights were inferior to equal-weights where there were fewer than 100 observations per predictor variable available for estimating the model [25]. Yet, many practical problems—including election forecasting—involve limited sample sizes.

For election forecasting, one study found that equal-weights versions of two published regression models provided out-of-sample election forecasts that were at least as accurate as those from the original regression models [26]. Another study showed that equal-weights versions of six of nine established regression models for election forecasting yielded more accurate forecasts than the original models. On average across the ten elections from 1976 to 2012, the equal-weights models reduced the original regression models’ *ex ante* absolute forecast errors by 5% [24].

### Combine forecasts from alternative models

Hundreds of studies have shown that combining forecasts that incorporate diverse data and information is an effective method for using additional knowledge and to thereby improve forecast accuracy [27].

Reviews of studies on combining forecasts conclude that simple unweighted averages provide the most accurate forecasts, except in rare situations where strong evidence suggests that some models consistently provide more accurate forecasts than others [28]. That paper also found that the error of simple unweighted averages of forecasts from six election-forecasting models was 25% lower than the corresponding error of the forecasts from a much more complex combining method. In light of the evidence, we calculated simple unweighted averages of the forecasts from all models with the same dependent variable to generate combined forecasts for this study.

### Use all important variables

Include all known important variables in a model. The guideline is difficult to implement with multiple regression analyses because the practical limit of the method is a handful of variables at best [29]. Researchers typically confront the problem by using only some of the variables that are known to be important.

One way to avoid the practical limits that regression places on the number of variables in a model is to use *prior knowledge* instead of statistical methods to select causal variables and determine their direction and size effects. This necessitates a review of the cumulative knowledge from prior research. Knowledge models can be traced back to a letter from Benjamin Franklin, in which he described “Moral Algebra, or Method of Deciding Doubtful Matters,” his method for choosing between alternatives [30]. In short, Franklin recommended identifying all important variables and whether they add to or subtract from the likelihood or value of the alternative. Next, weight each variable by the strength of its effect. Finally, apply the model you have just developed to each alternative by ascertaining the values of the variables, multiplying by the model’s assigned weights, and adding to obtain the score for the alternative model. A higher scoring alternative is more likely, or better.

The major advantage of this approach is that variables are included on the basis of prior knowledge about their importance (i.e., substantive effect) and direction, and not on the basis of a given set of data alone. Consequently, one does not need to estimate a coefficient for each variable from the data and the number of variables that can be included in a model is unlimited.

Franklin suggested differential weighting of variables. Forecasters, however, often lack adequate prior knowledge about the relative importance of important variables. Given the evidence on the relative accuracy of equal and regression weights outlined above, equal variable weights are a reasonable starting point for causal models. As the number of variables in a model increases, the magnitudes of individual variable effects become less important for predictive validity, as an early paper showed mathematically [31].

Franklin’s approach was intended for rating alternatives, but when the dependent variable is a scalar and data are available, the scores for alternatives can be used as the independent variable in single regression analysis. One study tested that approach by assigning equal-weights to all 27 (standardized) variables that were included in nine established models for forecasting U.S. presidential elections. The resulting model was used to generate *ex ante* forecasts of the ten elections from 1976 to 2012 with an average error of 1.3 percentage points. That error was 48% smaller than the typical model’s error and 29% smaller than the most accurate model’s error [24].

The present study uses a similar approach and sums the standardized values of all variables that are used in different models that predict the same target variable in order to calculate an index variable. The resulting vote equation is:

$$V=a+b\sum_{i=1}^{N}x_{i}$$

where the *x*_*i*_’s are the standardized values of *N* unique variables used in different models.

## Method

All data and calculations are available at the Harvard Dataserve: https://doi.org/10.7910/DVN/OI9IA3.

### Model estimation and forecast generation

For each of the 19 models, we standardized the original data and transformed variables to ensure that all predictor variables correlated positively with the dependent variable. Standardization of variable values was performed by calculating the differences from their mean and dividing by their standard deviation. Transformation for variables that are correlated negatively with the dependent variable was done by multiplying the variable values by -1.

We analyzed the accuracy of forecasts across all observations available for each model. All forecasts were out-of-sample using an N-1 cross-validation procedure, an approach that is also known as jackknifing. In other words, to forecast an election outcome we estimated models using the data on all other elections in the data set. This method allows for a powerful test of predictive validity because it maximizes both the size of the estimation sample and the number of out-of-sample forecasts.

All data and calculations are based on the models’ specifications published in the respective journal publications. Often, however, these versions were different from the original specifications that were used to predict a particular election. For example, Ray Fair changed his model equation in 1992, and kept it constant since [3]. Most models have been revised at least once since their first publication, usually as a reaction to poor performance in forecasting the previous election. Such revisions usually improve model fit, because the model developer has access to historical data when selecting the variables and building the model. One study showed that model accuracy drops substantially for observations not available at the time of model development [24].

In sum, N-1 cross-validation favors regression analysis in producing forecasts that use more information than one would have had available at the time of making the prediction. Hence, any accuracy gains from applying the conservative guidelines obtained in the present study should be regarded as a lower boundary.

### Error measure

We report the relative absolute error (RAE) of the forecasts that result from the application of each guideline [32]. The RAE is calculated as the mean absolute error (MAE) of forecasts from a model that follows the guideline, divided by the corresponding MAE of the original model. Values of RAE greater than 1 mean that following a guideline yielded forecasts that were *less* accurate than those from the original model, whereas values less than 1 mean that following the guidelines yielded *more* accurate forecasts.

## Accuracy gains from following Golden Rule guidelines

### Modification of estimated effects

#### Damping

Across all 19 models, only damping of 20% or less reduced errors for most models and on average, and the error reductions were small. For example, damping model coefficients by 10% reduced error for 14 of the 19 models (74%), with an average error reduction of 2% (= 1–0.98). Heavier damping than 20% harmed accuracy. Table 2 shows the mean RAEs of the forecasts across all 19 models with coefficients damped from 10% to 100% in intervals of 10%, while S1 Table in the supporting information shows the RAEs for forecasts from each individual model for each of the ten levels of damping.

**Table 2:** Effect of damping and equalizing on forecast errors relative to original forecast errors.

Level of equalizing / damping (%)	Damping		Equalizing	
	Mean RAE	% Mean RAEs < 1	Mean RAE	% Mean RAEs < 1
10	0.98	74	0.97	100
20	0.99	63	0.96	95
30	1.02	47	0.95	89
40	1.07	37	0.94	89
50	1.15	32	0.93	89
60	1.23	32	0.92	89
70	1.32	32	0.92	89
80	1.41	26	0.93	89
90	1.52	26	0.93	84
100	1.62	26	0.94	79
** Mean**	**1.23**	**39**	**0.94**	**89**

#### Equalizing

All levels of equalizing reduced forecast error on average. Error reductions ranged from 3% to 8%. Moreover, equalizing reduced the errors of forecasts from at least 15 of the 19 models for all levels of equalizing. The most extreme equalizing—in which all predictor variables are assigned equal-weights in the models—provided forecasts with a mean RAE of 0.94. In other words, equal-weights models reduced forecast error compared to forecasts from the original models by, on average, 6%. Table 2 shows the mean RAEs of the forecasts across all 19 models with equalizing from 10% to 100% in intervals of 10%, while S2 Table in the supporting information shows the RAEs for forecasts from each individual model for each of the 10 levels of equalizing.

Error reductions were maximized, more or less, with equalizing of 50% and, both mean RAEs and the percentage of models with RAEs of less than one improving little and then deteriorating with more equalizing. In sum, the results suggest that, by providing an efficient trade-off between average error reduction (RAE) and the chance of error reduction (% Mean RAEs < 1), 50% equalizing is a sensible compromise. Moreover, this 50–50 rule is easy to understand and easy to apply: simply average the forecast from the original regression model and the forecast from an equal-weights version of the model.

### Forecast combinations

The benefits of combining forecasts can be tested for elections for which (a) more than one model is available and (b) the models predict the same dependent variable. This was the case for the eight models that forecast U.S. presidential elections and the two models that forecast Australian general elections. (Note that although two models were available for predicting Canadian federal elections, those models predict a different outcome—incumbent party vote for one, and Liberal party vote for the other—and thus their forecasts could not be combined.) Table 3 shows the results.

**Table 3:** Effect of combining on forecast errors relative to original forecast errors.

	MAE (original)	RAE (combined)
***Australia*, *22 elections from 1951 to 2004***		
***MAE of combined forecast***	***2*.*26***	
Cameron & Crosby [5]	2.68	0.84
Jackman [6]	2.54	0.89
***Mean (typical model) error***	***2*.*61***	***0*.*86***
***U*.*S*., *15 elections from 1956 to 2012***		
***MAE of combined forecast***	***1*.*48***	
Abramowitz [17]	1.76	0.84
Campbell [18]	1.99	0.74
Cuzán [16]	2.07	0.72
Erikson & Wlezien [21]	2.54	0.58
Fair [3]	2.49	0.60
Holbrook [20]	2.55	0.58
Lewis-Beck & Tien [19]	2.29	0.65
Lockerbie [22]	2.73	0.54
***Mean (typical model) error***	***2*.*30***	***0*.*64***

For Australian elections, model forecasts were combined across the 22 elections from 1951 to 2004 for which forecasts from both models were available. The MAE of the combined forecast was 2.26 percentage points, which was more accurate than the forecasts from both of the individual models. Compared to the average model forecast (with an error of 2.61 percentage points), combining reduced error by 14%.

For U.S. elections, model forecasts were combined across the 15 elections from 1956 to 2012 for which forecasts from all eight models were available. The MAE of the combined forecast was 1.48 percentage points and was thus smaller than the average errors of each of the eight individual models, which ranged from 1.76 to 2.73 percentage points. Compared to the error of the typical model, which was 2.30 percentage points, combining reduced error by 36% (Table 3). The larger error reduction in the U.S. compared to Australia was expected as the combination included four times more models (eight versus two).

Compared to the error of forecasts from Abramowitz’s model, the RAE of the combined forecast was 0.84, which means that forecast combining reduced error by 16% compared to the single model that performed best in retrospect. Thus, even if one knew what would be the best model, it was better to use the combined forecast.

### Use more of the important variables: Knowledge models

Similar to the tests of combining forecasts, the benefits from using more important variables in one model could be tested only for U.S. and Australian elections. While the conservative guideline is to include *all important variables* in the forecasting model (a “knowledge model”), it is important to note that our test was limited to the variables from the respective countries’ election models. We would expect larger gains in accuracy when more of the relevant causal variables are included.

Table 4 shows the error reductions achieved by using all of the variables used by the experts in models that weight each of the variables equally. In the Australian case, the model included a total of six variables: the five variables used by Cameron & Crosby [5], plus one additional variable—a different measure of unemployment—used by Jackman [6]. The other two variables in the Jackman model, inflation and “honeymoon”, are also in the Cameron and Crosby model. Across the 22 elections, the “all-variables” model forecasts had an average error of 2.35 percentage points, which is lower than the error of each of the individual model forecasts. Compared to the typical model, the more-variables model reduced error by 10%, and 8% respectively compared to the best individual model.

**Table 4:** Effect of using all variables in an equal-weights knowledge model on forecast errors relative to original forecast errors.

	MAE (original model)	RAE (knowledge model)
**Australia (6-variable knowledge model),** **22 elections from 1951 to 2004**		
** MAE of knowledge model forecast**	**2.35**	
Cameron & Crosby [5]	2.68	0.88
Jackman [6]	2.54	0.92
***Mean (typical model) error***	***2*.*61***	***0*.*90***
**U.S., (24-variable knowledge model),** **15 elections from 1956 to 2012,**		
** MAE of knowledge model forecast**	**1.32**	
Abramowitz [17]	1.76	0.75
Campbell [18]	1.99	0.66
Cuzán [16]	2.07	0.64
Erikson & Wlezien [21]	2.54	0.52
Fair [3]	2.49	0.53
Holbrook [20]	2.55	0.52
Lewis-Beck & Tien [19]	2.29	0.58
Lockerbie [22]	2.73	0.48
***Mean (typical model) error***	***2*.*30***	***0*.*57***

In the U.S. case, the all-variables model included 24 variables. While the total number of variables used in the eight models is 28, four variables were excluded: The models of Fair [3] and Cuzán [16] use three identical variables, and Fair’s WWII dummy variable is unnecessary for our more-variables model since we only examine elections for which data for all eight models are available, from 1956 onwards. Across those 15 elections, the MAE of the all-variables model forecasts was 1.32 percentage points, which is lower than the errors of each of the individual models. Compared to forecasts from the typical model, the all-variable model reduced error by 43%. Compared to forecasts from the best individual model, the all-variable model reduced forecast error by 25%.

## Discussion

In this paper, we applied three conservative forecasting guidelines to 19 published regression models for forecasting election results. The guidelines were: (I) modify effect estimates to reflect uncertainty, (II) combine forecasts from dissimilar models, and (III) include all variables that are important in the model.

For the first guideline, we tested two approaches to modifying effect estimates to make them more conservative: damping and equalizing. Small levels of damping yielded 2% *ex ante* forecast error reductions, but higher levels harmed accuracy. Equalizing the regression coefficients almost always improved forecast accuracy and reduced *ex ante* forecast error by between 3% and 8% in comparison to the typical original model forecasts.

Armstrong, Green and Graefe suggested that the “optimal approach most likely lies in between… statistically optimal and equal, and so averaging the forecasts from an equal-weights model and a regression model is a sensible strategy” [1]. The evidence from the present paper supports that contention. Equalizing of 50%, which is equivalent to the suggested approach, reduced error for nine out of ten forecasts, with an average error reduction of 7%. In addition to the improved accuracy of the resulting forecasts, the 50–50 rule has other benefits: it is easy to understand, remember, and apply; simply average the forecast from the original regression model with the forecasts from an equal-weights version of the model.

Applying the second guideline—combining forecasts—to eight U.S. models, and to two Australian models, produced forecasts that were more accurate than those from the individual model that provided the most accurate forecasts in each case. Compared to the typical individual model forecast, error was reduced by 36% in the U.S. case and 14% in the Australian case. The results are thus consistent with the average of 22% error reduction for five comparative studies from different areas—including forecasts of economic variables—that examined combining across dissimilar causal models [1]. The results are also consistent with the guideline that forecasters should aim to include all important information in the forecast, rather than seeking to estimate statistically optimal effect sizes from historical data for a small set of selected variables. The “combine forecasts from dissimilar models” guideline is an established strategy for incorporating more information.

The third guideline recommends an alternative approach to incorporating more information into a forecast: to use all important variables in the one “knowledge model”. As with combining, knowledge models provided forecasts that were more accurate than even the best individual model. Compared to the typical forecast, a knowledge model that assigned equal-weights to all unique variables from the original published models reduced forecast error by 10% in the case of the six-variable Australian model and 43% in the case of the 24-variable U.S. model. As expected, including more variables that have an important causal relationship with the variable being forecast impoved forecast accuracy.

Our tests found that the strongest implementation of the conservative guidelines, in the form of knowledge models, provided the greatest improvement in *ex ante* forecast accuracy. That the knowledge models simply applied equal weights to standardized causal variables suggests that regression estimated weights contribute less to a model’s descriptive power, or realism, in practice than does including more of the variables that are known to be important.

Implementing the conservative guidelines offers more than simply improved *ex ante* forecast accuracy—as practically useful as that is. Knowledge models, for example, which include all important variables, also offer greater validity. First, the models are consistent with theory and knowledge *and* produce smaller forecast errors than competing models. Second, the models include more causal variables and thereby provide a more complete representation of domain knowledge. Forecasters who use knowledge models must have extensive domain knowledge in order to select all relevant variables and code the direction (and potentially the relative strengths) of their effects. Hence, they need to (i) study prior theories to identify which variables likely have an effect and (ii) rely on findings from experimental research. They should also (3) consult other experts to ensure that important knowledge has not been overlooked.

The gains from combining forecasts and from using more of the important variables were achieved for election forecasting models that, for the most part, used similar variables. We expect that further gains in accuracy and model realism could be achieved by incorporating variables that measure other important effects on voting, such as candidates’ prior experience [33] and their issue-handling competence and leadership skills [4].

Many forecasters are wary of incorporating a large number of variables into a model, regarding parsimony as an important quality of a forecasting model [34]. Models that use fewer variables likely put fewer demands on the forecaster than identifying and using all relevant knowledge and information. But is parsimony in the use of knowledge and information a good strategy in developing a forecasting model? Our findings suggest otherwise. Moreover, by assigning equal weights to variables, knowledge models are arguably *more* parsimonious than MRA models, because equal weights models need meet none of the many and onerous statistical assumptions that must be—but are rarely—met for regression analysis.

## Conclusions

The strict assumptions of regression analysis are seldom met in practice. As a consequence, the question of which method should be used for developing a forecasting model cannot be settled by asserting the superior statistical properties of an optimal regression model. Damping—for which the results were mixed—aside, the error reductions of between 3% and 43% found in the study reported in this paper support the contention that for practical forecasting problems, models developed by following conservative forecasting guidelines are likely to provide forecasts that are more accurate than those from the original econometric models.

Forecasters who value forecast accuracy should endeavor to include all important variables in a model. The variables should be assumed to be equally important in the absence of prior experimental evidence.

The gains in accuracy reported in this paper were achieved for election forecasting, a problem that involves little uncertainty and only modest complexity. Larger gains in forecast accuracy might be possible when the Golden Rule of Forecasting guidelines are applied to complex problems that involve much uncertainty. Such problems include forecasting election outcomes in more volatile political jurisdictions, but also less-structured problems, such as forecasting the onset of political conflicts, the costs and benefits of government policies, and the long-term economic growth of nations. Further empirical studies on the value of applying the Golden Rule of Forecasting to such problems would help to assess the conditions under which the guidelines improve accuracy.

## Supporting information

S1 Table Relative absolute error (RAE) of forecasts from damping compared to forecasts from the original regression models. (DOCX)

S2 Table Relative absolute error (RAE) of forecasts from equalizing compared to forecasts from the original regression models. (DOCX)
