# Supplementary material for: Accuracy gains from conservative forecasting: Tests using variations of 19 econometric models to predict 154 elections in 10 countries
Source: PLoS One. 2019 Jan 10;14(1):e0209850. doi: 10.1371/journal.pone.0209850 (PMC6328130; doi:10.1371/journal.pone.0209850)
Supplement: S2 Table — (DOCX) [file pone.0209850.s002.docx]

|  | **MAE** | **RAE depending on equalizing factor (in %)** | | | | | | | | | |
| --- | --- | --- | --- | --- | --- | --- | --- | --- | --- | --- | --- |
| **Model** | **0%** | **10** | **20** | **30** | **40** | **50** | **60** | **70** | **80** | **90** | **100** |
| Abramowitz (16) | 1.69 | 0.99 | 0.98 | 0.98 | 0.97 | 0.96 | 0.95 | 0.95 | 0.94 | 0.93 | 0.92 |
| Campbell (17) | 1.97 | 0.99 | 0.99 | 0.98 | 0.98 | 0.97 | 0.96 | 0.96 | 0.95 | 0.95 | 0.94 |
| Cuzán (15) | 2.05 | 0.98 | 0.96 | 0.95 | 0.93 | 0.91 | 0.90 | 0.89 | 0.89 | 0.89 | 0.90 |
| Bellucci (8) | 2.21 | 0.97 | 0.94 | 0.91 | 0.88 | 0.85 | 0.81 | 0.78 | 0.75 | 0.72 | 0.69 |
| Lewis-Beck & Tien, US (18) | 2.25 | 1.00 | 0.99 | 0.99 | 0.98 | 0.98 | 0.98 | 0.97 | 0.98 | 0.99 | 1.00 |
| Lewis-Beck. Nadeau & Bélanger (14) | 2.33 | 0.98 | 0.95 | 0.93 | 0.91 | 0.89 | 0.86 | 0.84 | 0.82 | 0.80 | 0.77 |
| Erikson & Wlezien (20) | 2.47 | 0.88 | 0.99 | 0.98 | 0.98 | 0.97 | 0.96 | 0.96 | 0.95 | 0.94 | 0.94 |
| Jackman (5) | 2.54 | 0.99 | 0.98 | 0.97 | 0.96 | 0.96 | 0.95 | 0.95 | 0.95 | 0.96 | 0.96 |
| Holbrook (19) | 2.59 | 0.99 | 0.98 | 0.96 | 0.95 | 0.95 | 0.95 | 0.97 | 0.99 | 1.01 | 1.03 |
| Lockerbie (21) | 2.73 | 0.99 | 0.99 | 0.98 | 0.97 | 0.97 | 0.96 | 0.96 | 0.95 | 0.94 | 0.94 |
| Fair (3) | 2.80 | 0.95 | 0.90 | 0.86 | 0.83 | 0.80 | 0.77 | 0.77 | 0.78 | 0.79 | 0.80 |
| Magalhães & Aguiar-Conraria (11) | 3.17 | 0.95 | 0.94 | 1.02 | 1.10 | 1.18 | 1.27 | 1.40 | 1.54 | 1.68 | 1.82 |
| Cameron & Crosby (4) | 4.22 | 0.97 | 0.94 | 0.92 | 0.89 | 0.87 | 0.86 | 0.85 | 0.85 | 0.84 | 0.84 |
| Dassonneville, Lewis-Beck & Mongrain (10) | 4.34 | 1.00 | 1.00 | 1.00 | 1.02 | 1.03 | 1.05 | 1.09 | 1.12 | 1.16 | 1.20 |
| Bélanger & Godbout (6) | 4.59 | 0.98 | 0.96 | 0.95 | 0.93 | 0.91 | 0.89 | 0.88 | 0.86 | 0.84 | 0.82 |
| Lewis-Beck & Tien, JP (9) | 4.79 | 0.99 | 0.97 | 0.96 | 0.94 | 0.93 | 0.92 | 0.90 | 0.89 | 0.88 | 0.87 |
| Nadeau & Blais (7) | 7.01 | 0.97 | 0.94 | 0.91 | 0.88 | 0.86 | 0.83 | 0.81 | 0.79 | 0.77 | 0.76 |
| Magalhães, Aguiar-Conraria, Lewis-Beck (12) | 9.86 | 0.96 | 0.93 | 0.89 | 0.86 | 0.82 | 0.79 | 0.75 | 0.72 | 0.69 | 0.66 |
| Toros (13) | 12.44 | 0.97 | 0.94 | 0.91 | 0.87 | 0.86 | 0.87 | 0.88 | 0.88 | 0.89 | 0.90 |
| **Mean** | **4.00** | **0.97** | **0.96** | **0.95** | **0.94** | **0.93** | **0.92** | **0.92** | **0.93** | **0.93** | **0.94** |
| **Notes:**   - Models are ordered from most to least accurate by mean absolute error (MAE) of the original individual regression models’ out-of-sample error (determined by N-1 cross validation) across all available observations. - The MAEs are the percentage point error that one would achieve without any (i.e., 0%) equalizing. | | | | | | | | | | | |
